# A model-based approach to predict muscle synergies using optimization: application to feedback control

**DOI:** 10.3389/fncom.2015.00121

**Published:** 2015-10-06

**Authors:** Reza Sharif Razavian, Naser Mehrabi, John McPhee

**Affiliations:** Department of Systems Design Engineering, University of WaterlooWaterloo, ON, Canada

**Keywords:** muscle synergy, real-time control, model-based approach, optimization, operational space, task-specific, dynamic redundancy, unique solution

## Abstract

This paper presents a new model-based method to define muscle synergies. Unlike the conventional factorization approach, which extracts synergies from electromyographic data, the proposed method employs a biomechanical model and formally defines the synergies as the solution of an optimal control problem. As a result, the number of required synergies is directly related to the dimensions of the operational space. The estimated synergies are posture-dependent, which correlate well with the results of standard factorization methods. Two examples are used to showcase this method: a two-dimensional forearm model, and a three-dimensional driver arm model. It has been shown here that the synergies need to be task-specific (i.e., they are defined for the specific operational spaces: the elbow angle and the steering wheel angle in the two systems). This functional definition of synergies results in a low-dimensional control space, in which every force in the operational space is accurately created by a unique combination of synergies. As such, there is no need for extra criteria (e.g., minimizing effort) in the process of motion control. This approach is motivated by the need for fast and bio-plausible feedback control of musculoskeletal systems, and can have important implications in engineering, motor control, and biomechanics.

## 1. Introduction

The human musculoskeletal system has a redundant structure—there are more degrees of freedom than required to perform a certain task (kinematic redundancy), and each degree of freedom is actuated by multiple muscles (dynamic redundancy). These redundancies make the control problem challenging. Humans usually take this ability for granted, without noticing the complexities involved.

Muscle synergy has been proposed as a possible strategy to reduce the dimensions of the control space (the number of variables modulated by the nervous system) in the control of musculoskeletal systems (for a short review see Tresch and Jarc, 2009). According to this hypothesis, the central nervous system (CNS) activates a group of muscles together; within each group, the muscles are activated via fixed patterns. Therefore, instead of activating all the muscles individually, the CNS combines a far fewer number of bundles of activation to build the required muscle forces. There are, however, important questions that need to be answered regarding the plausibility of this theory.

### 1.1. Structure and number of synergies

The structure of the dimension reduction in the nervous system via muscle synergies is not properly understood. *Muscle synergy* is often defined as fixed relations between instantaneous activation levels of multiple muscles (Ting, 2007; McKay and Ting, 2008; Berniker et al., 2009; Roh et al., 2011; Safavynia et al., 2011; Kutch and Valero-Cuevas, 2012; Steele et al., 2013; Zelik et al., 2014). Alternatively, time-varying patterns (also called the *motor primitives*) are proposed as the building blocks of muscle activations (D'Avella and Tresch, 2001; Ivanenko et al., 2006; Bizzi et al., 2008; d'Avella et al., 2008; Sartori et al., 2013). A mixture of both approaches has also been investigated by Delis et al. (2014).

The identification of the synergies is an important part of the theory. Various methods have been proposed in the literature to decompose muscle activities into a number of synergies. In the majority of research articles, the goal has been to reconstruct the measured muscle activities as closely as possible, using a low-dimensional basis set (the synergies). Non-negative matrix factorization (NNMF, Lee and Seung, 2000; Sharif Shourijeh et al., 2015a) is a widely-used method in this application (d'Avella et al., 2008; McKay and Ting, 2008; Berniker et al., 2009; Kargo et al., 2010; Berger and d'Avella, 2014). This approach, however, is unable to determine whether the synergies result from neural origins (as claimed by the synergy theory), or are by-products of other processes [e.g., biomechanical constraints (Kutch et al., 2008; Kutch and Valero-Cuevas, 2012), or optimization (de Rugy et al., 2013)].

There is also uncertainty about the number of synergies. The usual practice is to examine the variance accounted for (VAF) of the experimental EMG after synergy decomposition (Lockhart and Ting, 2007; Roh et al., 2011; de Rugy et al., 2013; Moghadam et al., 2013; Sartori et al., 2013; Steele et al., 2013; Delis et al., 2014). In general, a fewer number of synergies produce a lower VAF, and as the number of synergies increase, more variation in the experimental data can be captured. Therefore, the number of synergies beyond which no further improvement in VAF is observed is usually chosen. Unfortunately this approach is purely statistical, and does not provide significant insight into biomechanical aspects of muscle synergy theory.

### 1.2. Dependency of the synergies on the task and posture

The dependency of synergies on the task and posture has not been extensively investigated. Efforts have been made to find *shared* synergies that can reconstruct EMG data in a variety of tasks (e.g., Bizzi et al., 2008; Sartori et al., 2013; Zelik et al., 2014). In the majority of the articles, however, synergies from a single task are studied, without explicit investigation as to if the synergies vary from one task to another. de Rugy (2010) has shown that visuomotor adaptation occurs at the muscle synergy level, suggesting the necessity of task-dependent synergies. It is, therefore, reasonable to argue that the recruited set of synergies may depend on the intended action. For example, the set of synergies used during a hand-writing action is perhaps different from the set recruited during a simple grasp motion, though the same muscle are activated. Our hypothesis is that different sets of synergies are known to the CNS, and the CNS chooses the appropriate set to manipulate and perform the tasks.

Therefore, information about the intended task seems to be essential in the identification of the synergies. For this purpose, a quantifiable criterion is needed to distinguish between tasks. We hypothesize that the desired controlled variable (or the operational space) could provide such information. For example, in a point-to-point reaching task, Morasso (1981) found that the hand position and velocity follow a stereotypical trajectory (straight line motion with bell-shaped velocity profile). These findings suggest that the hand position is the actively controlled variable, and the operational space is the two-dimensional Cartesian space for hand position. On the contrary, in an elbow flexion/extension task, joint angle and angular velocities follow such stereotypical trajectories, meaning that the controlled variable, rather than being hand position, is the joint angle (i.e., the operational space is the one-dimensional joint angle space, rather than a two-dimensional Cartesian space for hand position). Scholz and Schöner (1999) have presented the *uncontrolled manifold theory* to systematically identify the controlled variable in various tasks. According to this theory, the variability is higher in the dimensions irrelevant to the intended task than those directly related to it. This theory aligns well with the *minimal intervention theory*, which states that the CNS activates muscles to control only the task-relevant variable (Valero-Cuevas et al., 2009). Both theories support our hypothesis that the synergies, if they exist, have a direct relation with the intended task.

The effects of posture on synergies also needs to be studied. d'Avella et al. (2008) proposed tonic and phasic synergies for gravity balancing and acceleration, respectively. They were able to estimate the tonic synergy coefficients based on the final posture, and the phasic coefficients based on the velocity, using cosine tuning curves. We would like to expand the idea of posture-dependent synergies (by defining them based on the operational space variables), and study the usefulness of these synergies in motion control.

### 1.3. Functional aspects of the synergies

Most synergy analysis processes in the literature only involve EMG reconstruction. Few studies have used synergy decomposition while taking into account the reconstruction of force/torque in the operational space. de Rugy et al. (2013) and Moghadam et al. (2013) identified synergies corresponding to various directions of wrist force and shoulder torque, respectively. Nonetheless, de Rugy et al. (2013) found high levels of error in the force reconstruction if too few synergies were used.

Using synergies to control motion is another challenge. Feedback control of musculoskeletal systems that act on task space variables is a appealing; however, the literature is limited (Lockhart and Ting, 2007; Ting, 2007), in which the center of mass position was used as the feedback to construct a balance controller.

More articles are available regarding the application of muscle synergy in the feed-forward control of motions (for example McKay and Ting, 2008; Berniker et al., 2009; Neptune et al., 2009; Kargo et al., 2010; Allen and Neptune, 2012). However, these studies reported that the synergies need to be fine-tuned, which is inherent to feed-forward control. Furthermore, despite the reduction in the number of control inputs, the problem is still redundant, requiring an optimization routine to solve for the best combination of the synergies.

### 1.4. Relation to optimal control

There is a tight relation between muscle synergy theory and optimal control of motion. Essentially, an optimal pattern of muscle activities is inherently synergistic (de Rugy et al., 2013; Steele et al., 2013). We also observe that the output of the nervous system shows signs of optimality, meaning that if synergies do exist, they are optimal. We, therefore, hypothesize that the muscle synergy and optimality are interrelated—we show that synergies can be defined directly through optimization. To support this approach, we argue that the results of the optimization process (perhaps in course of human evolution) may have been learned and stored in the nervous system as synergies (see the discussion in de Rugy et al., 2012). A high-level controller (e.g., a robust or an optimal controller in Todorov and Jordan, 2002; Todorov et al., 2005) can then employ the synergies for the control of actions.

### 1.5. Relation of the current work with the existing literature

The following work addresses some of the aforementioned issues about muscle synergy theory via mathematical modeling and optimization. The novel contribution of this paper is the introduction of a model-based approach for the identification of muscle synergies, as an alternative to the factorization methods (in which the synergies are extracted from EMG data). It has been previously mentioned that synergies may arise from a background optimization process (either on-line optimization or evolutionary adaptation, de Rugy et al., 2012, 2013); however, no formal mathematical argument has been provided in the literature.

We also propose that the number of synergies depends on the intended task (i.e., the number of dimensions of the operational space). As a result of our model-based approach, the number of synergies is determined by the requirements of the musculoskeletal system and the task, resolving the discrepancy regarding the number of synergies in the literature. Furthermore, by examining the operational space, it is possible to distinguish between seemingly similar tasks, which may require substantially different synergies. The task-specific definition of the synergies is yet another novel contribution of our work that has not been previously investigated.

Lastly, these synergies simplify the redundant force-sharing problem in the musculoskeletal system, resulting in a unique solution for the muscle activities. Uniqueness of the solution is a fundamental feature of dimension reduction in motor control that has not been properly addressed. As a result, a simple feedback control scheme can be constructed without the need to solve an on-line optimization problem. This idea resembles the hierarchical control framework in Todorov and Jordan (2002) and Todorov et al. (2005); however, their relation to muscle synergy is less explicit. Such a fast and bio-plausible control scheme has significant implications in various fields, including faster simulation of musculoskeletal systems, predictive forward analysis of motion, prosthetic and orthotic device design, rehabilitation, and Functional Electrical Stimulation of muscles.

## 2. Materials and methods

In this section, we will present the basics of our muscle synergy framework for the control of musculoskeletal systems. Previous studies show that optimization-based solutions to the muscle force-sharing problem results in realistic muscle activation patterns (for a review see Erdemir et al., 2007). Therefore, we have based our mathematical arguments on optimization results.

Muscle synergies will be defined based on the task, and for a certain operational space. For example, if the operational space (the controlled variable) is the elbow flexion angle, one flexor and one extensor synergies are needed. However, in reaching actions where the operational space is the two-dimensional (2D) position of the hand, flexion/extension synergies are irrelevant, and the shoulder and elbow muscles are recruited to satisfy the 2D hand force requirements. We hypothesize that multiple sets of synergies are known to the CNS, and different sets are recruited during different tasks.

Two examples are provided to showcase our methodology; a 2D one-degree-of-freedom (one-DoF) musculoskeletal forearm model (Sharif Shourijeh and McPhee, 2013; Sharif Razavian et al., 2015) has been used to explain the mathematical foundation of the method. Then, the method is generalized to a more complex three-dimensional (3D) human driver model (Mehrabi et al., 2015a,b). Although differences exist in the aforementioned tasks and recruited muscles, since the operational space in both systems is one-dimensional, our method can define two posture-dependent synergies that sufficiently control the motion.

### 2.1. Synergistic control of a simple model

**Figure 1:**
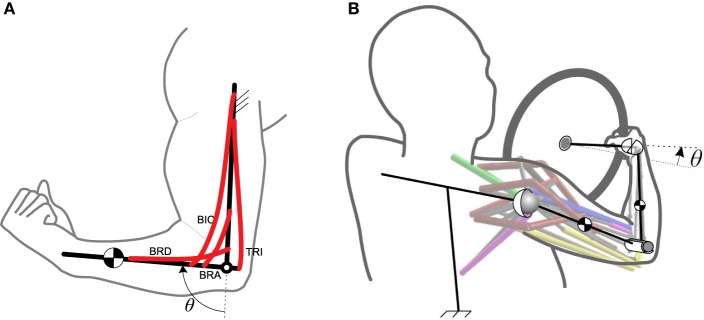
**The musculoskeletal models. (A)** The 2D forearm model. **(B)** The 3D driver model. Both models are one-DoF, but the operational spaces are different.

The simple musculoskeletal forearm model used to introduce our muscle synergy framework is shown in Figure 1A. This model consists of seven muscles: brachioradialis, brachialis, biceps brachii (long and short heads), and triceps brachii (long, lateral, and medial heads). The physical parameters for these muscles are taken from Sharif Shourijeh and McPhee (2013). The model has one DoF at the elbow joint (flexion/extension angle, θ), which is considered as the operational space.

Since the model contains only mono-articular muscles, it is possible to analytically solve for the optimal muscle activations, *a*_*i*_, that minimize the instantaneous cost function *J*:

(1) $$J=\sum_{i=1}^{m}a_{i}^{2}\;(m=\text{number of muscles})$$

subject to the constraints:

(2) $$\sum_{i}F_{i}r_{i}(\theta )=T$$

and

(3) $$0\leq a_{i}$$

The cost function *J* in Equation (1) represents the muscular effort at each instant of time. Equation (2) is the moment balancing constraint that requires the muscles to generate a certain torque *T* in the operational space. The muscle forces, *F*_*i*_, act at posture-dependent moment arms *r*_*i*_(θ), which are positive for the elbow flexors (i.e., brachioradialis, brachialis, and the two biceps brachii heads), and negative for the extensors (triceps brachii heads).

The inequality constraint (Equation 3) enforces the activations to be positive. No explicit upper bound (i.e., *a*_*i*_ ≤ 1) is assumed for the activations since lower activations are strictly preferred by the cost function, resulting in optimal activations that do not violate the upper bound constraint. Therefore, our arguments are only valid for *sub-maximal* motions.

The muscle force can be estimated from the activation level using a Hill muscle model as:

(4) $$F=aF_{0_{max}}f_{l}(\theta )f_{v}(\dot{\theta },\;a)\cos(\alpha )$$

In the Hill muscle model, muscle activation, *a*, scales the maximum muscle force *F*_0_*max*__. Additionally *f*_*l*_ and *f*_*v*_ are force-length and force-velocity relations (Thelen, 2003) that also alter the muscle force. Lastly, muscle force in the tendon direction is affected by the pennation angle α.

**Figure 2:**
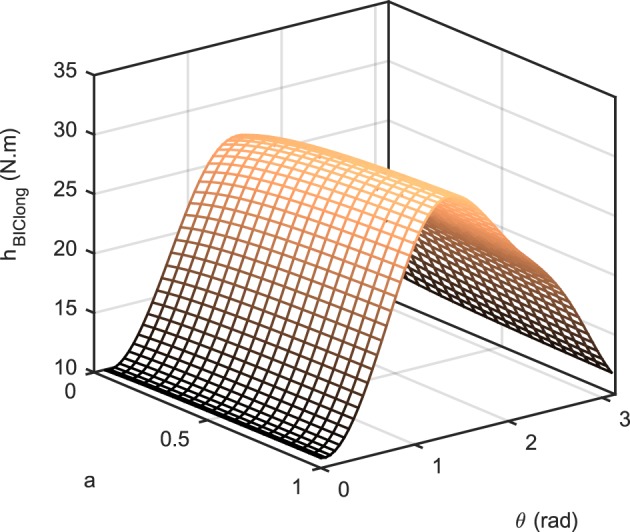
**The transformation ***h***_***BIClong***_ as a function of joint angle θ and activation ***a***, for $\dot{\theta }=2$ rad/s**.

We can combine the non-linear terms in Equations (2, 4) and rewrite the constraint (Equation 2) as:

(5) $$\sum_{i}a_{i}h_{i}(\theta ,\;\dot{\theta })=T$$

where $h_{i}\left(\theta ,\dot{\theta }\right)$ is the non-linear function that transforms muscle activity to the torque in the operational space (similar to a Jacobian that transforms joint torque to end-effector force); it accounts for the force-length relation *f*_*l*_, force-velocity relation *f*_*v*_, maximum force *F*_0_*max*__, pennation angle α, and moment arm *r*(θ) of muscle *i*. Therefore, *h* is positive for the flexors and negative for the extensors. It should be noted that in Equation (5), we have neglected the dependency of the force-velocity term on the activation [i.e., $f_{v}=f_{v}\left(\dot{\theta }\right)$]. Figure 2 shows the value of *h* for the long head of biceps brachii as a function of joint angle θ and activation for $\dot{\theta }=2$ rad/s. As can be seen, *h* is not a significant function of activation.

Solving this optimization problem (details given in the Appendix) yields the optimal activations:

(6) $$\begin{array}{l} a_{i}^{*}=0\\ \text{or} \end{array}$$

(7) $$a_{i}^{*}=\frac{h_{i}(\theta ,\;\dot{\theta })}{\sum_{j}h_{j}^{2}(\theta ,\;\dot{\theta })}T$$

The optimal solutions (Equations 6, 7) are both valid answers in different situations. When the joint torque *T* is positive, the solution (Equation 7) is valid for the flexor muscles, which have $h\left(\theta ,\dot{\theta }\right)>0$. For the extensors, however, *h* is negative resulting in a negative (infeasible) answer if Equation (7) is used. Therefore, the optimal extensor activations when *T* > 0 are stated by Equation (6) (i.e., no extensor activity.) The opposite argument can be made when the joint torque is negative. In this case, the optimal extensor activations are found using Equation (7), while flexors are inactive^1^. Therefore, the closed-form solution of Equation (7) can be used to efficiently calculate the optimal muscle activations that generate a certain joint torque, *T*.

Alternatively, we can observe that the ratio of the activations for the same-action muscles is independent of the required torque; they all activate with fixed (posture-dependent) relations—the same notion as muscle synergy.

(8) $$\frac{a_{i}^{*}}{a_{j}^{*}}=\frac{h_{i}}{h_{j}}=f(\theta ,\;\dot{\theta })$$

It is possible to define two *synergies* for this operational space: one for a positive joint torque (flexor, *S*^*f*^), and one for a negative one (extensor, *S*^*e*^). We can identify two representative muscles (i.e., a flexor and an extensor) from the full set of muscles, and calculate the *synergy ratios* of Equations (9, 10).

(9) $$S_{i}^{f}=\{\begin{array}{ll} \frac{a_{i}^{*}}{a_{f}^{*}}=\frac{h_{i}}{h_{f}} & S_{i}^{f}>0\\ 0 & S_{i}^{f}\leq 0 \end{array}$$

(10) $$S_{i}^{e}=\{\begin{array}{ll} \frac{a_{i}^{*}}{a_{e}^{*}}=\frac{h_{i}}{h_{e}} & S_{i}^{e}>0\\ 0 & S_{i}^{e}\leq 0 \end{array}$$

In these relations, $S_{i}^{f}$ and $S_{i}^{e}$ are the flexor and extensor synergy ratios for muscle *i*, respectively. We can calculate the optimal muscle activation for muscle *i* based on the flexor and extensor representatives (*a*_*f*_, *a*_*e*_) using:

(11) $$a_{i}=a_{f}\;S_{i}^{f}+a_{e}\;S_{i}^{e}$$

The representative activations themselves can be calculated either from the optimal values (Equation 7), or from any other control logic. It is important to note that, regardless of the values of (*a*_*f*_, *a*_*e*_), if the synergy ratios of Equations (9, 10) are used, the resulting torque is optimally produced.

**Figure 3:**
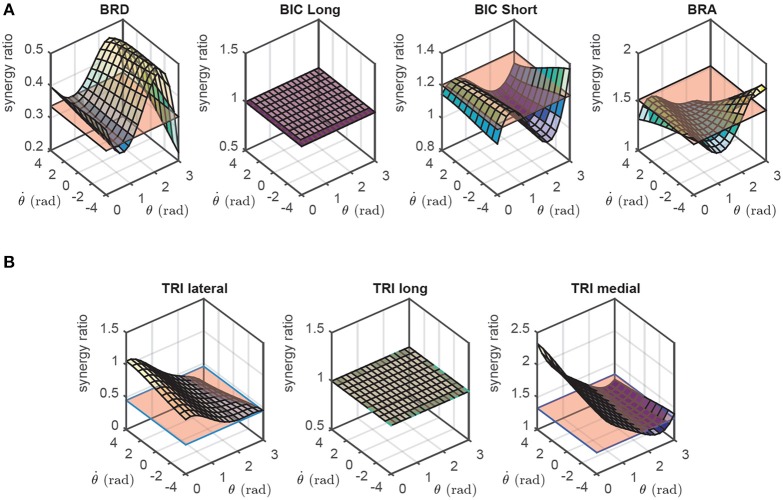
**The flexor (A) and extensor (B) synergy ratios in the 2D model as functions of the joint angle (θ) and angular velocity ($\dot{\theta }$)**. These synergies are compared against the static synergies resulting from NNMF algorithm (the flat surfaces, see Section 2.3).

The calculation of synergy ratios are straightforward in this model; they are the ratio of non-linear transformation of muscle *i* to that of the representative muscle. Although *h* is in general a function of activation, we can safely neglect such dependency and calculate *h*|_*a* = 0.5_. As shown later, this approach results in near-optimal solutions. The flexor and extensor synergy ratios for the 2D forearm model are shown in Figure 3, where the long head of biceps and the long head of triceps are chosen as the representative flexor and extensor muscles, respectively. It should be noted that the choice of the flexor and extensor representatives are arbitrary in this model because the muscles have explicit flexor/extensor functions.

### 2.2. Synergistic control of the 3D arm

As a more complex example, we have considered a 3D arm model rotating a steering wheel, Mehrabi et al. (2015a,b, see Figure 1B). This model consists of four body segments: trunk, upper arm, forearm, and hand. The trunk is assumed to be fixed, and the upper arm is attached to the trunk using a spherical joint. The elbow is modeled as a revolute joint, and the hand is connected to the forearm via a universal joint. Since it is assumed that the hand grips the steering wheel firmly, the whole system has only one DoF. Therefore, knowing the steering wheel angle is sufficient to find the arm joint angles. This argument is not valid in general, as there is one extra DoF (supination/pronation) that is neglected for the sake of simplicity. In the case that this extra DoF exists, we will need more synergies to control the motion, which is out of the scope of this paper.

The objective function is still the minimization of muscular effort (Equation 1). However, the operational space in this model is no longer the joint angle; instead, the desired operational space (i.e., the variable that is controlled) is the steering wheel angle.

For this complex system of driver/steering wheel, the mathematical arguments similar to Equations (1–7) are more difficult to make. However, since the model has only one DoF, it is possible to generalize the arguments to accommodate this 3D arm as well.

Given the complex kinematics in this model, direct solution for *h* (as used in Equation 5) is challenging. An efficient method is to calculate it from the response of musculoskeletal system similar to the experimental procedure in Berger and d'Avella (2014). At a certain posture, activation of each muscle will produce a torque in the operational space (in this case steering rotation θ). We can define the non-linear transformation *h*(θ) as:

(12) $$h_{i}(\theta )≜\frac{T_{i}}{a_{i}}$$

where *a*_*i*_ is the activation of muscle *i* and *T*_*i*_ is the resulting torque in the operational space. Having *h* calculated from Equation (12), it is now possible to use the constraint of Equation (5), thereby making similar arguments to calculate the optimal activations and the synergy ratios:

(13) $$S_{i}^{cw}=\{\begin{array}{ll} \frac{a_{i}^{*}}{a_{cw}^{*}}=\frac{h_{i}}{h_{cw}} & S_{i}^{cw}>0\\ 0 & S_{i}^{cw}<0 \end{array}$$

(14) $$S_{i}^{ccw}=\{\begin{array}{ll} \frac{a_{i}^{*}}{a_{ccw}^{*}}=\frac{h_{i}}{h_{ccw}} & S_{i}^{ccw}>0\\ 0 & S_{i}^{ccw}<0 \end{array}$$

These synergy ratios are calculated based on two representative muscle activations: a counter-clockwise rotator and a clockwise rotator, which are denoted by the subscripts *cw* and *ccw*, respectively.

In general, *h* is a function of both the steering angle and angular velocity. However, as previously shown in the 2D results (Figure 3), *h* and synergy ratios, *S*, are not significantly affected by $\dot{\theta }$. Therefore, we assume that *h* is only a function of θ; i.e., *h* = *h*(θ). This assumption significantly reduces the complexity of the synergies and the memory required to store them. However, it comes at the expense of slight sub-optimality if the synergy ratios of Equations (13, 14) are used (we will show later that they are close to optimal). Furthermore, such an assumption aligns well with the concept of posture-dependent synergies, whereas, velocity-dependency has not been reported before.

**Figure 4:**
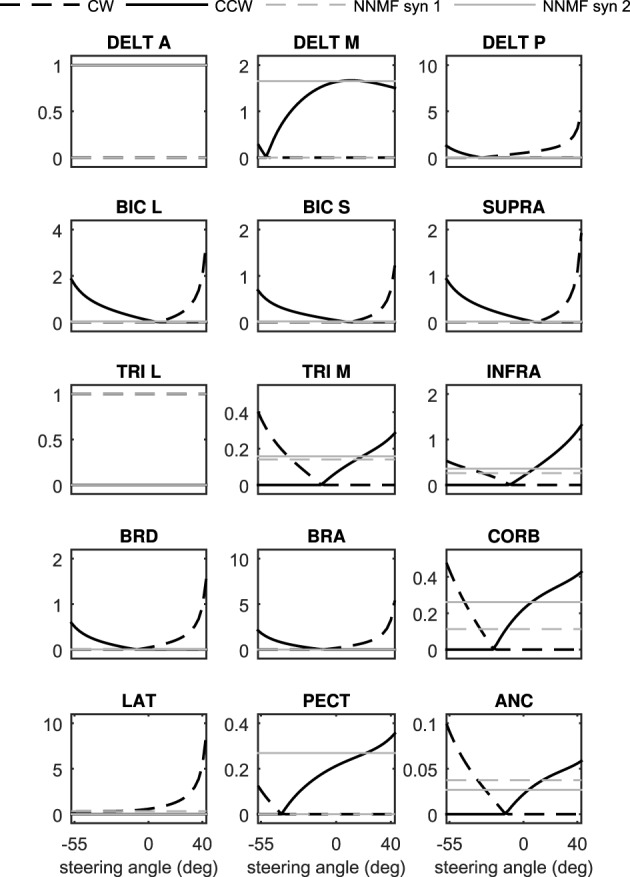
**The posture-dependent synergy ratios, ***S***_***i***_, as functions of steering angle**. They are compared against the static NNMF synergies (the gray constant lines).

To summarize the procedure, we can activate any muscle *i* individually at a certain posture, measure the resulting torque, and then calculate *h*_*i*_(θ) using Equation (12). Doing the same procedure for all muscles and at various postures will result in a set of posture-dependent *h*_*i*_(θ), which in turn can be used to calculate the synergy ratios from Equations (13, 14). Figure 4 shows the synergy ratios for all 15 muscles in this model, where latissimus dorsi (Jonsson and Jonsson, 1975) and anterior deltoid (Hayama et al., 2012) are the clockwise and counter-clockwise representatives, respectively. It is interesting to note that except for three muscles (anterior deltoid, long head of triceps, and latissimus dorsi) all others change function at a certain steering wheel angle (from CCW rotator to CW rotator or vice versa). This phenomenon limits us to chose any arbitrary muscles as the representatives. This observation also highlights the necessity of synergy dependency on posture.

### 2.3. Comparison with non-negative matrix factorization

The established method to extract the synergies usually involves the generation of a large matrix containing all the EMG data, which is then fed to a factorization algorithm. The most widely used algorithm in this context is Non-Negative Matrix Factorization (NNMF Lee and Seung, 2000). The NNMF decomposes the original EMG data matrix, **A**, into two matrices: the non-negative synergy matrix, **S**, and the non-negative coefficient matrix, **C** as:

(15) $$A_{m\times l}=S_{m\times n}C_{n\times l}$$

where *m* is the number of muscles, *l* is the number of samples, and *n* is the number of synergies. Each column of the synergy matrix **S** represents a synergy, and contains the relative contributions of each muscle in that synergy. A row in the coefficient matrix, **C**, contains the activation level of the corresponding synergy for all the samples.

The samples in the data matrix may vary based on the experiment; they can be snapshots of the time-varying muscle activities, or the average of the recodings from multiple trials. Regardless, NNMF results in synergies that are essentially static—i.e., they are the same for all samples.

It has been shown in Steele et al. (2013) that one obtains similar synergies from NNMF with experimental EMG data as from NNMF with optimal activations. Therefore, the standard method of extracting synergies from the EMG data can be replaced by applying NNMF to the optimal muscle activations. Consequently, to compare our method with NNMF results, we have used the synergies extracted from optimal muscle activations as the benchmark. Figures 3, 4 show the synergies extracted using NNMF. To obtain these synergies, the optimal muscle activities were found such that the musculoskeletal systems followed a random motion in their operational space. These time-varying optimal muscle activities were gathered in the matrix **A**, and fed to the NNMF algorithm to find the static synergies. The calculated synergies were scaled so that the activation of the *representative* muscle would equal to unity.

As can be seen in Figures 3, 4, the NNMF synergies are close to the average of the posture-dependent synergies; however, because of the limited number of synergies (*n* = 2 in these cases), NNMF is unable to capture all such variations. As a result, the synergies resulting from NNMF are not suitable for for control purposes (see section Results).

### 2.4. Feedback control of musculoskeletal systems

The major motivation for this work is the need for a fast and bio-plausible feedback controller for the musculoskeletal system. The usual practice of optimization for the control of a musculoskeletal system is a time-consuming process, and cannot be used for real-time applications. It is also unrealistic to assume that the CNS can perform this amount of computations in real-time (de Rugy et al., 2012). The definition of muscle synergy presented in this work yields a unique solution for the force-sharing problem, thereby eliminating the need for any on-line optimization, resulting in a significantly faster feedback control scheme.

With the synergy ratios calculated beforehand, we can control the musculoskeletal system in an optimal manner by calculating only the representative muscle activations. All other muscle activations can optimally be constructed using the synergy ratios:

(16) $$\text{for the 2D model}:\;a=S^{f}\;a_{bic}+S^{e}a_{tri}$$

(17) $$\text{for the 3D model}:\;a=S^{ccw}\;a_{delt}+S^{cw}\;a_{Lat}$$

where **S**^*f*^, **S**^*e*^, **S**^*ccw*^, and **S**^*cw*^ are vectors containing all the synergy ratios, $S_{i}^{f}$, $S_{i}^{e}$, $S_{i}^{ccw}$, and $S_{i}^{cw}$, respectively.

The representative muscle activations can be found with various control methods such as forward static optimization (FSO), optimal control (e.g., model predictive controller, MPC, or linear quadratic regulator, LQR), or even a simple proportional-integral-derivative (PID) controller.

**Figure 5:**
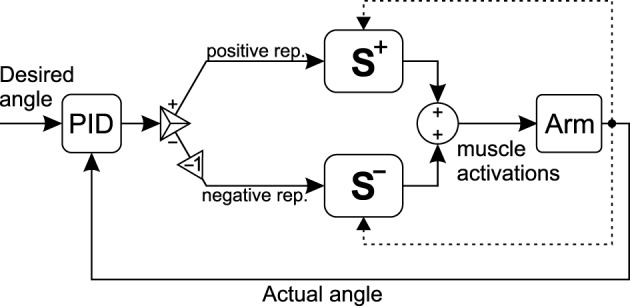
**The schematic of the control loop**. The output of the controller is a signed activation. The positive and negative portions of the signal are used to create muscle activations from the two synergy ratios.

To show the effectiveness of the synergies for real-time control, a simple PID controller is used to control the musculoskeletal systems (Figure 5). The output of the controller is a *signed activation*. Therefore, the positive and negative portions of the signal should be separated. The positive values are interpreted as the representative muscle activation for the flexor or CCW synergies, for the 2D and 3D models, respectively. Similarly, the negative portion is interpreted as the extensor/CW representative in the two models. The representative activations can subsequently be multiplied by the corresponding synergy ratios Equations (16, 17) to calculate all muscle activations.

## 3. Results

The simulation results for both the 2D and 3D models are presented here. In these simulations, the objective was to efficiently follow a desired trajectory in the operational space.

Two feedback control methods were used: an optimal controller [forward static optimization (FSO), Sharif Shourijeh et al., 2015b], and the PID controller. FSO was selected as our optimal controller because of its feedback properties and the fact that it results in optimal behavior (Anderson and Pandy, 2001). For the FSO controller, we considered a weighted sum of the muscular effort and tracking error as the objective function. The weighting factors and the PID controller parameters are provided in Table 1.

(18) $$J=w_{1}\sum_{i=1}^{m}a_{i}^{2}+w_{2}(\theta -\theta_{des})+w_{3}(\dot{\theta }-{\dot{\theta }}_{des})$$

**Table 1:** **Numerical values of the parameters used in the two simulations**.

**Parameter**		**2D model**	**3D model**
FSO	*w*_1_	1	1
	*w*_2_	3 × 10^6^	1 × 10^4^
	*w*_3_	5 × 10^2^	1 × 10^2^
PID	*K*_*p*_	10	100
	*K*_*i*_	10	100
	*K*_*d*_	2	0

Two sets of simulations were run. First, the musculoskeletal systems were driven by the optimal controller, resulting in our *gold standard* muscle activation patterns. In these simulations, the activation level of all the muscles were individually modulated by the FSO controller for each time step. In the second and third sets of simulations, the PID controller calculated the *signed* activation signal based on the tracking error (i.e., the difference between the desired and actual angle), which was used to construct the muscle activity levels using muscle synergies (Equations 16, 17). These muscle activities were used to drive the musculoskeletal systems.

**Figure 6:**
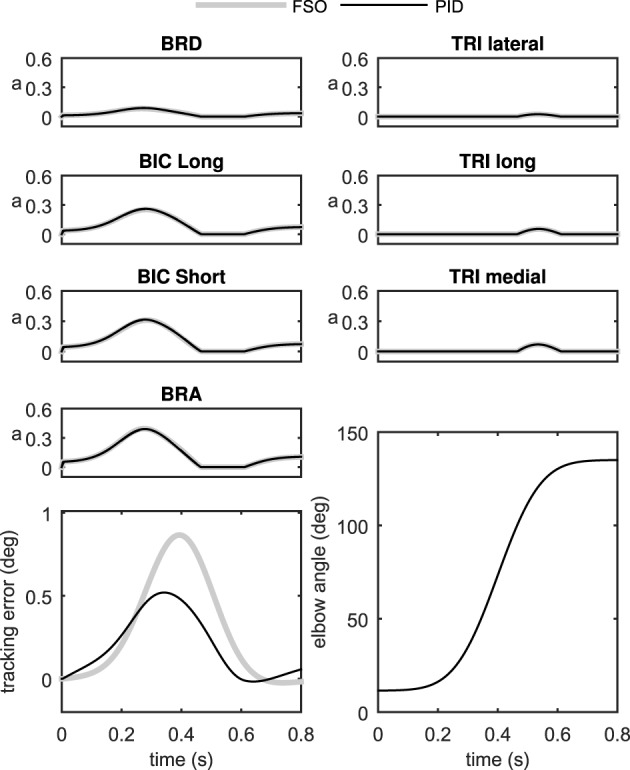
**Comparison of the two control methods in the control of the 2D forearm model**.

Figure 6 shows the performance of the two controller methods for the 2D model. As can be seen in this figure, the performance of the two controllers is very close. The tracking error is comparable using the two controllers, and the muscle activation patterns are also very similar.

The similarity of the activations resulting from the synergistic controller (PID) and the optimal controller (FSO) suggests that the synergies defined in the previous sections result in near-optimal behavior. The numerical values of the physiological cost (Equation 19) in Table 2 further show the closeness of the two methods. Previous reports (Erdemir et al., 2007) have shown that the optimal muscle activities (calculated by the FSO controller) estimate realistic muscle activities, which implies that our synergistic controller results in realistic activity patterns.

(19) $$\text{effort}=\frac{1}{T_{f}}\sum_{i}\int_{0}^{T_{f}}a_{i}^{2}dt$$

**Table 2:** **Comparison of the two control methods**.

**Method**	**Physiological effort**		**Computation time**^†^	
	**2D forearm model**	**3D driver model**	**2D forearm model**	**3D driver model**
FSO (baseline)	7.35 × 10^−2^	2.24 × 10^−1^	26.50 s	136.0 s ^‡^
PID (posture-dependent)	7.41 × 10^−2^	2.28 × 10^−1^	24.30 × 10^−3^ s	2.24 s ^‡^
PID (NNMF)	–	2.52 × 10^−1^	–	2.24 s ^‡^

† *Simulations on a 3.60 GHz quad-core Intel CPU with 16 Gb of RAM*.

‡ *Total simulation time, which includes the time required for controller calculations, plus the integration time of the musculoskeletal model*.

**Figure 7:**
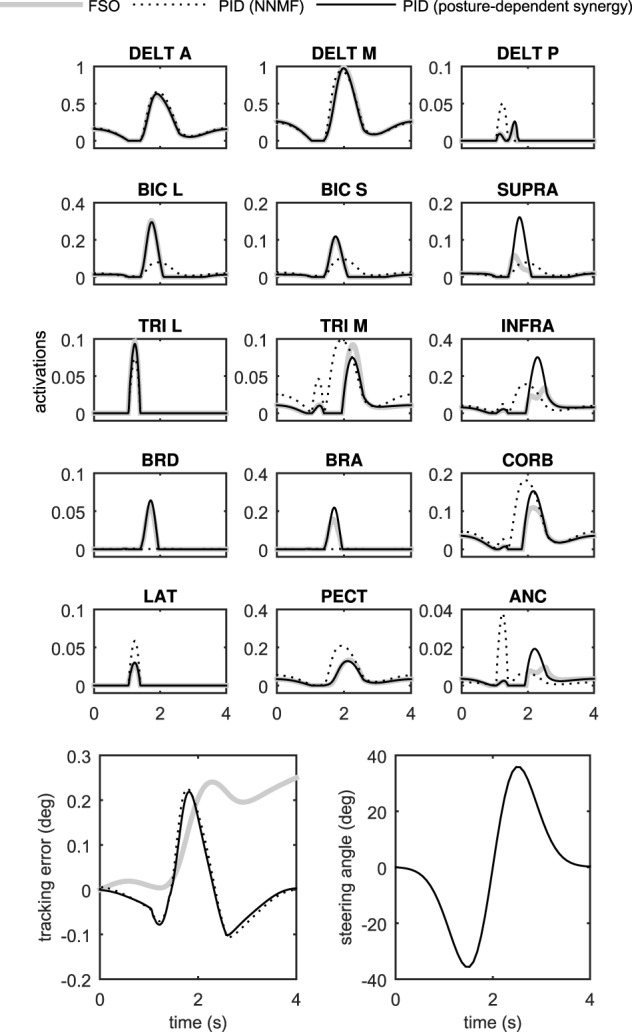
**Comparison of the two control methods in the control of the 3D driver model**.

Figure 7 presents the 3D model simulation results, which contains an extra set of simulations to compare the NNMF synergies with our posture-dependent synergies. Similar to the 2D model results, the optimal muscle activities are well-matched by the synergies presented in this paper. However, the static synergies from NNMF could not properly recreate the optimal (gold standard) muscle activities. This happens because the NNMF essentially averages the relative muscle activities for the entire range of motion in the operational space, therefore neglecting the changing importance and function of the muscles. As a result, some muscles are over-activated (e.g., medial head of triceps), incorrectly activated (e.g., posterior deltoid) or even completely neglected (e.g., brachioradialis and brachialis). Our definition of synergies allows for high reconstruction accuracy with the minimum number of synergies (in these cases only two synergies). The comparison of the numerical values of the physiological cost (Table 2) further show that the two NNMF synergies cannot reconstruct the optimal muscle activities as well as the posture-dependent ones (the physiological cost increases by 12% using NNMF synergies).

The synergistic controller performed similar to the optimal controller, but was 2–3 orders of magnitude faster (Table 2). These results show that the synergistic controller can run in real-time, which is an important requirement in many applications including real-time control of functional electrical stimulation and rehabilitation devices (see Section Discussion).

## 4. Discussion

Muscle synergy has been considered as a possible mechanism employed by the human nervous system to control movements. Previous investigations on muscle synergy usually relied on an *inverse* extraction method—i.e., the synergies were extracted from the measured muscle activities using a factorization algorithm (e.g., NNMF, Lee and Seung, 2000). These methods usually neglect the functional aspects of synergies and their correspondence with the task.

Unlike previous research, we proposed a model-based approach to define the synergies based on the principles of optimality. It has been argued that muscle synergies may arise from a background optimization process (perhaps during evolution) (de Rugy et al., 2012, 2013). Our method relies on these arguments, and defines the synergies by employing optimization tools. However, unlike the on-line optimization methods, e.g., (Todorov and Jordan, 2002; Todorov et al., 2005), the synergies are optimally calculated and stored off-line, and recalled during an on-line control process. In support of our method, it has been reported that the muscle activities estimated by optimal control approaches correlate well with experimental EMG (Erdemir et al., 2007), and that the synergies extracted from such optimal muscle activities match the ones extracted from the EMG data (Steele et al., 2013). The comparison between our results and the synergies obtained from the common factorization methods (NNMF) also show the plausibility of the optimal arguments. Therefore, the presented method can be used as a theoretical model-based framework to study muscle synergy—a tool that was not available before.

Another distinguishing feature of our approach is the dependency of the synergies on the posture and the task. To the best of our knowledge (and perhaps because of the vast number of the required experimental trials) no explicit definition of posture-dependent synergies is available in the literature. Our approach excels as it relates the synergies to known biomechanical parameters (such as muscle strength and moment arm, which are already available in the literature, e.g., Garner and Pandy, 2001). The posture dependency, as mentioned earlier in this paper and in de Rugy et al. (2012), might be an important requirement of synergies, as muscles may change function depending on the posture [e.g., wrist muscles (Kakei et al., 1999)]. Our results comparing the posture-dependent synergies and the fixed ones from NNMF support our hypothesis that posture-dependent synergies can reduce the dimensions of the control space more effectively; fewer synergies are required to efficiently control the motion if synergies are posture-dependent.

There are two schools of thought regarding the relationship between synergies and tasks: some researchers try to find the *shared* synergies that can explain muscle activities in a variety of motions (e.g., Bizzi et al., 2008; Sartori et al., 2013), while others look at specific tasks [e.g., point-to-point reach, (d'Avella et al., 2008), or wrist articulation, (de Rugy et al., 2013)]. Task-dependent synergies have been previously mentioned (e.g., in Zelik et al., 2014), but no scientific method to distinguish the tasks and relate the synergies to the operational space has been shown. We argue that for the efficient control of a task, it is essential for the CNS to recruit the synergies related to that specific operational space.

This argument immediately raises questions about how the CNS may learn and recall these synergies for every task. One possible argument is that the synergies (especially the ones related to locomotion) are fine-tuned over the course of human evolution, and perhaps hard-coded into the spinal cord circuitries (the so-called central pattern generators (Ijspeert, 2008) can be viewed as an example). Alternatively, and especially in the context of adaptation to new tasks, the synergies may be viewed as flexible structures, decoded by the interneurons of spinal cord (similar to the concept of *spinal-like regulators* proposed by Raphael et al., 2010). It has been shown in de Rugy (2010) that the process of visuomotor adaptation likely happens at the sensory level as well as the execution (muscle synergy) level. In the light of their results, we can argue that in a novel task (e.g., a distorted operational space), the previously learned synergies may not be able to span the new space (due to the highly non-linear transformations from the synergy space to the operational space); thus, the CNS needs to *learn* new synergies to effectively maneuver in the new operational space. It is likely that during the learning process, the CNS uses the previously known synergies as a starting point, and by trial and error develops a new basis set that is *good enough* (Loeb, 2012) to maneuver in the new task space.

### 4.1. Application to higher degrees of freedom: insights from robotics

**Figure 8:**
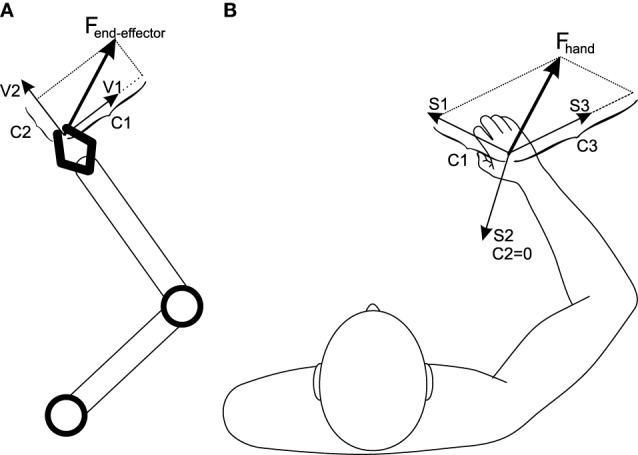
**(A)** A non-redundant robotic arm. The operational space is spanned by the basis set *V*_*i*_; an arbitrary force can be decomposed into this basis set, resulting in the required actuator efforts. **(B)** The human musculoskeletal system. Since the muscles are pull-only actuators, one extra basis vector is required to satisfy positive-decomposition constraint. The basis set, *S*_*i*_, in this case are synergy-produced forces, which can be used to decompose any arbitrary hand force.

The dependency of the number of synergies on the operational space dimensions can be explained from a mechanical point of view. Assume an *n*-DoF robot (Figure 8A) with an *n*-dimensional operational space; also assume that the robot is non-redundant (i.e., there are *n* actuators). In a certain state of the robot, each actuator can produces a force in the operational space (denoted by the vectors *V*_*i*_ in Figure 8A). The set of all *n* force vectors can be viewed as a basis set that spans the robot's operational space. To control the robot in its operational space, a required end-effector force can be decomposed onto the basis set, resulting in the decomposition coefficients *C*_*i*_. These coefficients correspond to each actuator effort (Khatib, 1987).

The human musculoskeletal system (Figure 8B) is different from a robot in two ways: it is actuated by muscles (they can only pull), and is also redundant (there are more actuators than the degrees of freedom).

The pull-only condition introduces the constraint that the end-effector force vector has to be positively-decomposed (i.e., the coefficients *C*_*i*_ must be positive). To positively-decompose an arbitrary vector in an *n*-dimensional vector space, *n* + 1 basis vectors are needed (instead of *n*), meaning that *n* + 1 pull-only actuators are needed.

The redundancy poses the challenge of non-uniqueness of the solution—the number of muscles is usually larger than *n* + 1. In order to reduce the redundant system to a non-redundant one, multiple muscle has to be grouped into *n* + 1 synergies; this way, each synergy's pulling direction can be used as a basis vector vectors (*S*_*i*_, *i* ∈ {1…*n* + 1} in Figure 8B) to span the operational space (this is essentially the same concept as the *cosine tuning curves* mentioned before e.g., in de Rugy et al., 2013).

The CNS can therefore, control the redundant musculoskeletal system by employing the *best* combination of muscle activities that generate such basis vectors in each posture. We argue that these *best sets* (or muscle synergies) are known to the CNS, and the CNS can reach a unique solution for the intensity of each synergy to generate a certain end-effector force, and consequently control the motion.

Our results show that when the operational space is one-dimensional, two posture-dependent synergies are enough to generate the motion. As the dimensions of the operational space increases, more synergies will be required; for instance, to control two dimensional point-to-point reaching action, three synergies are required so that any arbitrary hand force is positively-decomposed onto the synergies basis set. This hypothesis is supported by independent experimental analysis of reaching action in d'Avella et al. (2008), that three synergies can account for most of the variation in arm muscles EMG.

One important drawback of the application of the same method to higher operational space dimensions is the possible sub-optimality due to the force decomposition mechanism mentioned above. Although each basis vector is optimally produced by a single synergy, there is no guarantee that a linear combination of two synergies (to create an arbitrary force in the operational space) will remain optimal. Our one-DoF results were indeed optimal, because the operational space was always aligned with the optimally produced basis vectors. Our informal studies of higher-dimension systems show that the sub-optimality exist (although not significant). A possible strategy might be to increase the number of synergies. With more synergies the basis set is more packed, leaving smaller area to be spanned by two adjacent basis vectors. This strategy has been reported before in de Rugy (2010) where eight synergies were used to reconstruct six muscle activities. One immediate advantage of this *reversed dimension reduction* is the elimination of the need for optimization.

### 4.2. Other implications of the approach

The muscle synergy framework presented in this paper has important implications in different areas. Our approach to muscle synergy proposes answers to some unresolved issues in motor control studies, namely the number of synergies and their dependency on the task. In this paper we have presented ideas on the requirements of the synergies from a theoretical dynamics perspective. However, the reader should note that the results only show an initial study, and further experimental and/or theoretical investigations are necessary to make a stronger argument.

The results are even more interesting from an engineering perspective. The human musculoskeletal system is challenging to control due to the redundancy and non-linearities involved. Our muscle synergy approach introduces a way to simplify the control of such systems, which can be used in both simulations and real-life applications. Having a realistic controller that mimics the CNS behavior is a necessary component in predictive musculoskeletal simulations. Such a controller can generate/correct motions in unknown situations (e.g., in the presence of disturbances or when experimental motion is not available) without the need for computationally-intensive optimization solutions. It can also facilitate the design and control of machines interacting with humans, such as prosthetic and orthotic devices, exoskeletons, and rehabilitation robots (Ghannadi et al., 2015), by allowing fast prediction of the human behavior. Furthermore, the synergy controller can have direct application to the feedback control of real musculoskeletal systems via neurostimulation and functional electrical stimulation, where optimality and computational efficiency are absolute necessities.

## 5. Conclusion

In this paper, we presented a model-based mathematical method to define muscle synergies based on mathematical modeling and optimal control theory. We showed that muscle synergies can be effectively used to control a musculoskeletal arm in real-time. Using this approach, the indeterminate force-sharing problem in musculoskeletal system dynamics reduces such that the solution is unique. Our novel definition of the posture-dependent synergies allowed us to optimally generate torques in the operational space. This lent itself to both fast and efficient feedback control for the musculoskeletal systems. Our results showed that the difference in muscle activities and tracking performance between the feedback controller and the optimal results are insignificant, while the computations are ~1000 times faster with the former method. Further improvements can be made, however, by introducing a closed-loop control logic that takes into account predictive and learning properties of the human motor control system.

## Conflict of interest statement

The authors declare that the research was conducted in the absence of any commercial or financial relationships that could be construed as a potential conflict of interest.

## Footnotes

^1^A subtle detail that need to be considered in Equation (7) is that, depending on which group is active, the summation in the denominator has to be calculated over the *same-action* muscles (either flexors, or extensors).

## Appendix

To solve the optimization problem, the cost function (Equation 1) can be augmented using a Lagrange multiplier, λ (Kirk, 2004).

(A1) $$\hat{J}=\sum_{i}a_{i}^{2}+\lambda \left[\sum_{i}a_{i}h_{i}(\theta ,\;\dot{\theta })-T\right]$$

The inequality constraints (Equation 3) can be rewritten using the slack variables *s*_*i*_ in the form:

(A2) $$a_{i}-s_{i}^{2}=0$$

where *s*_*i*_ are assumed to be unbounded. Substituting Equation (A2) into Equation (A1) yields:

(A3) $$\hat{J}=\sum_{i}s_{i}^{4}+\lambda \left[\sum_{i}s_{i}^{2}h_{i}(\theta ,\;\dot{\theta })-T\right]$$

(A4) $$\sum_{i}s_{i}^{2}h_{i}(\theta ,\;\dot{\theta })=T$$

At a local minimum, the gradient of the cost function should be zero.

(A5) $$\frac{\partial \hat{J}}{\partial s_{i}}=4s_{i}^{3}+2\lambda s_{i}h_{i}(\theta ,\;\dot{\theta })=0$$

One answer to this equation is:

(A6) $$s_{i}=0\Rightarrow a_{i}^{*}=0$$

If *s*_*i*_ ≠ 0, we can divide (Equation A5) by *s*_*i*_ to get:

(A7) $$2s_{i}^{2}+\lambda h_{i}(\theta ,\;\dot{\theta })=0$$

which leads to:

(A8) $$s_{i}^{2}=-\frac{\lambda }{2}h_{i}(\theta ,\;\dot{\theta })$$

By substituting this expression into the constraints (Equation A4), the Lagrange multiplier can be found:

(A9) $$\sum_{i}\left[-\frac{\lambda }{2}h_{i}(\theta ,\;\dot{\theta })\right]\;h_{i}(\theta ,\;\dot{\theta })=T$$

(A10) $$\lambda =\frac{-2T}{\sum_{i}h_{i}^{2}(\theta ,\;\dot{\theta })}$$

Therefore, the optimal solution (in sub-maximal contractions) can be found:

(A11) $$s_{i}^{2}=a_{i}^{*}=\frac{h_{i}(\theta ,\;\dot{\theta })}{\sum_{j}h_{j}^{2}(\theta ,\;\dot{\theta })}T$$

### Nomenclature

**Table:**

α	Muscle pennation angle
Ĵ	Augmented cost function
λ	Lagrange multiplier in optimization
**A**	Data matrix
**C**	Coefficient matrix
**S**	Synergy matrix
θ	Angle in the operational space
*a*	Muscle activation
*a*^*^	Optimal muscle activation level
*F*	Muscle force
*f*_*l*_	Muscle force-length relation
*f*_*v*_	Muscle force-velocity relation
*F*_0_*max*__	Maximum isometric muscle force
*h*	Non-linear transformation from muscle activity to operational space torque
*J*	Cost function in optimization
*K*_{*p, i, d*}_	PID parameters
*l*	Number of samples in the NNMF data matrix
*m*	Number of muscles in the models
*n*	Number of synergies in NNMF algorithm
*r*	Muscle moment arm
*S*	Synergy ratio
*s*	Slack variable in optimization
*T*	Torque in the operational space
*w*	Weighting factor in objective function
